# A Fatal Bacteremia Caused by Hypermucousviscous KPC-2 Producing Extensively Drug-Resistant K64-ST11 *Klebsiella pneumoniae* in Brazil

**DOI:** 10.3389/fmed.2018.00265

**Published:** 2018-09-21

**Authors:** Tatiana Amabile de Campos, Laura Fernandes Gonçalves, Kelly Grace Magalhães, Vicente de Paulo Martins, Georgios Joannis Pappas Júnior, Gisele Peirano, Johann D. D. Pitout, Guilherme Bartolomeu Gonçalves, João Pedro Rueda Furlan, Eliana Guedes Stehling, André Pitondo-Silva

**Affiliations:** ^1^Department of Cell Biology, Institute of Biological Sciences, University of Brasilia, Brasília, Brazil; ^2^Department of Pathology and Laboratory Medicine, University of Calgary, Calgary, AB, Canada; ^3^Department of Clinical, Toxicological and Bromatological Analysis, School of Pharmaceutical Sciences of Ribeirão Preto, University of São Paulo, Ribeirão Preto, Brazil; ^4^School of Dentistry, University of Ribeirão Preto, Ribeirão Preto, Brazil

**Keywords:** *Klebsiella pneumoniae*, hypermucousviscous, KPC-2, XDR, ST11, virulence, antimicrobial resistance

## Abstract

We report a fatal bacteremia caused by *Klebsiella pneumoniae* in a 60–70-year-old patient from Brazil. The genomic analysis of three isolates (from blood culture, nasal and anal swabs) showed that the bacteremia was caused by a KPC-2 producing extensively drug-resistant K64-ST11 hypermucousviscous *K. pneumoniae* (hmKP) harboring several virulence and antimicrobial resistance genes. Although the isolates did not present virulence markers associated with hypervirulent *K. pneumoniae* (hvKP), they showed invasion and toxicity to epithelial Hep-2 cells; resistance to cell microbicidal mechanisms; and blood and human serum survival, evidencing their pathogenic potential. This study highlights the risk of infection caused by hmKp strains not characterized as hvKP as well as the clinical implications and difficulty of treatment, especially in elderly or immunocompromised patients.

## Introduction

### Case presentation

A 60–70-year-old patient, presenting a transverse colon neoplasm, underwent a left hemicolectomy at a tertiary hospital located in Brazil. For 9 months prior to surgery, the patient presented severe diarrhea (five to six episodes a day) and weight loss (14 kg). At anesthetic induction, the patient received venous ciprofloxacin, metronidazole, and tazobactam. Four days later, after discharge from the intensive care unit (ICU), he presented acute kidney injury, systemic arterial hypertension, chronic atrial flutter and suspected septic shock. Explorative laparoscopy showed anastomotic dehiscence and enteric fluid in the peritoneal cavity. The patient was promptly transferred back to the ICU. A blood culture was requested and returned a negative result. At this time, meropenem and amphotericin B were administered as antimicrobial therapy. Two days later, infectious surveillance and dialysis were initiated. For 4 days the patient remained non-febrile, clinically stable and septic shock was considered resolved. Therefore, amphotericin B was discontinued and meropenem was maintained as antibiotic therapy. Five days later, the patient had a cardiac arrest followed by a bradycardia episode. A cardiopulmonary resuscitation with noradrenaline was performed with a positive response. The patient was submitted to mechanical ventilation, and subsequently developed a pulmonary purulent infection in the orotracheal tube. Polymyxin B was introduced as antimicrobial therapy. Eighteen days after hospitalization, the patient showed deterioration in general health, presented bradycardia (not atropine reversible), and eventually died due to cardiac arrest.

### Bacterial isolates

Seventeen days after patient hospitalization, blood, anal and nasal swabs were collected and all three bacterial cultures were positive for *K. pneumoniae* by the VITEK-2 system (bioMerieux Brasil) (KpBSB-A, KpBSB-B, and KpBSB-C). All isolates showed the hypermucoviscosity phenotype determined by the string test (1).

### Bacterial survival in blood and serum

All isolates were used to evaluate their capacity for survival in human blood and serum (2). For this approach, bacterial suspensions previously grown at 37°C in Luria-Bertani (LB) medium for 18 h, were incubated in 550 μL of human blood (10^7^ bacterial cells) and in 550 μL human serum (10^8^ bacterial cells) at 37°C. Aliquots of 10 μL from each inoculum were removed at times 0, 30, 60, and 120 min and plated in Agar MacConkey medium (Kasvi). All plates were incubated at 37°C for 18 h for colony forming unit (CFU) recovery quantification. For the assays, blood and serum were collected from five health donors. All isolates were tested in triplicate for each donor.

**Figure 1:**
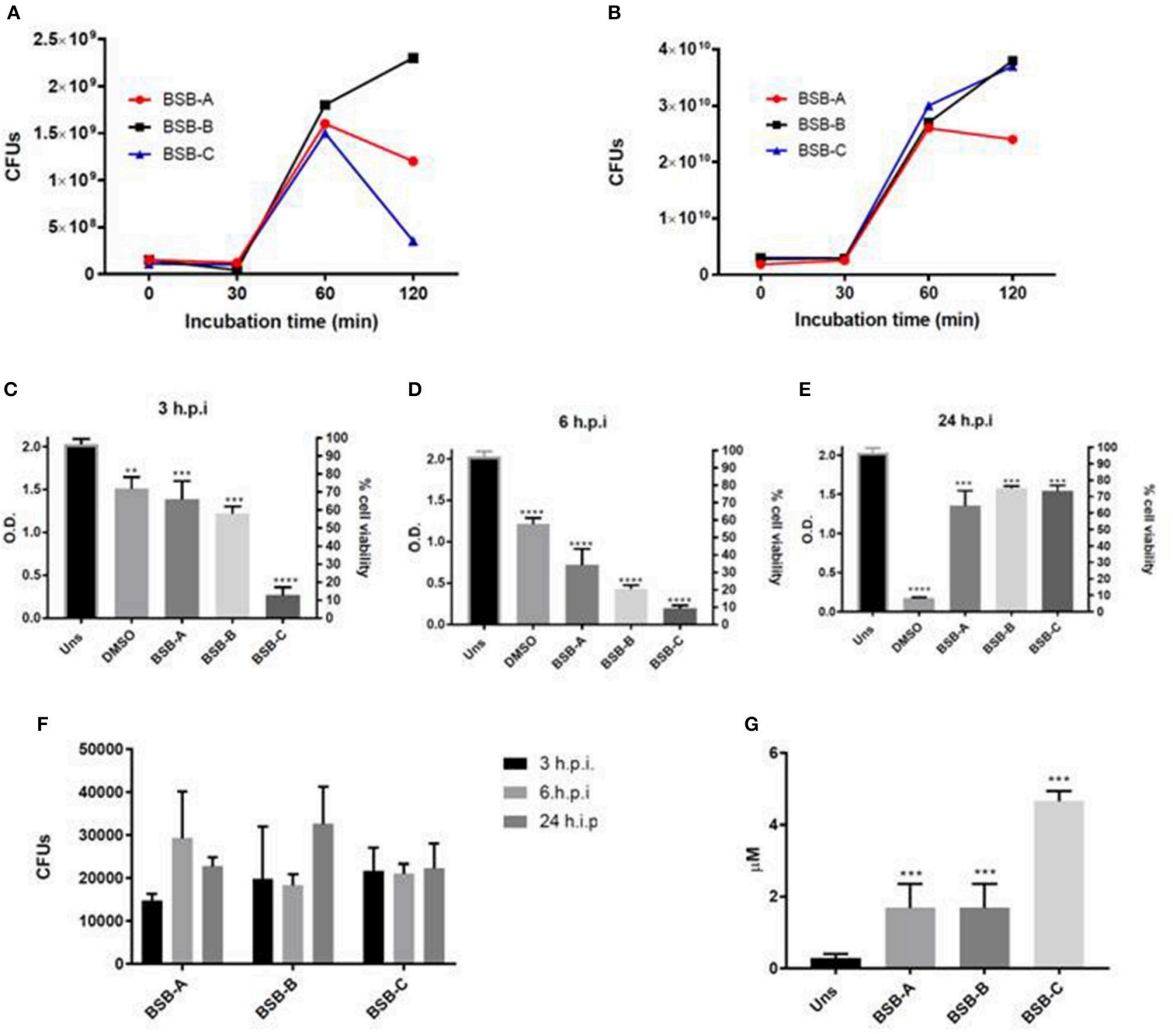
Biological characterization of the three hypermucousviscous *Klebsiella pneumoniae* isolates (BSB-A; BSB-B, BSB-C). **(A)** Bacterial blood survival evaluation of CFUs (colony forming units) recovery after isolate incubation in blood for 30, 60, and 120 min. **(B)** Bacterial human serum survival evaluation of CFUs recovery after isolate incubation in serum for 30, 60, and 120 min. **(C–E)** Hep-2 cells viability after different periods of infection by *K. pneumoniae* isolates. **(F)** Bacterial invasion of Hep-2 cells by *K. pneumoniae* isolates evaluated by CFUs recovery after 3, 6, and 24 h of infection. (**G)** Nitric Oxide quantification in Hep-2 cells after 24 h of infection by *K. pneumonia* isolates. Uns: Unstimulated cells (not submitted to bacterial infection). Control: unstimulated cells (not submitted to bacterial infection). DMSO: positive control for citotoxicity. ^*^p < 0.05; ^**^p < 0.01; ^***^p < 0.001. All infected cells were compared to unstimulated cells (non infected cells).

The results showed that the CFU recovery increased 2 times (2x) after 30 min of incubation in blood and serum. After 60 min, the recovery decreased in blood for isolates BSB-A and BSB-C, and increased for BSB-B (Figure 1A). All isolates showed an increase in CFU count after 60 min of incubation, and two of them (BSB-A and BSB-C) after 120 min of incubation in human serum (Figure 1B). These results indicate that the isolates were able to survive and grow in human serum and blood.

### Infection of Hep-2 cells by bacterial isolates

Bacterial invasion, cytotoxicity, resistance to epithelial cells and microbicidal mechanisms were evaluated with Hep-2 cells which were infected by the isolates with multiplicity of infection 1:1,000 (Hep-2 cells: isolates) as described by Favre-Bonte *et al*. (3). Following infection, the Hep-2 cells were incubated at 37°C and 5% CO_2_. After 30 min, the cells were washed with phosphate-buffered saline and the DMEM (Dulbecco's Modified Eagle's medium) (Gibco) medium was replaced by DMEM with 10 mg/mL of Gentamicin (Sigma Aldrich). Bacterial invasion and intracellular survival were evaluated by CFU quantification at 3, 6, and 24 h post infection (h.p.i.). Bacterial cytotoxicity was determined by MTT (3-(4, 5-dimethylthiazolyl-2)-2, 5-diphenyltetrazolium bromide) assays (Sigma Aldrich) at 3, 6, 24, and 48 h.p.i; and Hep-2 microbicidal mechanisms by nitric oxide (NO) quantification using Griess reagent (Promega) 24 h.p.i. All assays were carried out in triplicate for all isolates.

All isolates showed cytotoxic activity by reduced Hep-2 viability at 3, 6, and 24 h.p.i. (Figures 1C–E). Interestingly, after 24 h.p.i., all Hep-2 infected cells recovered their cell viability. About 15,000–20,000 CFUs were recovered from cells infected by the three isolates at 3 h.p.i. (Figure 1F). For cells infected by KpBSB-A, CFU recovery increased about 2x at 6 h.p.i (30,000) and 0.5x at 24 h.p.i. (2,000) (Figure 1F). For cells infected by KpBSB-B, CFU recovery remains at about 15,000 at 6 h.p.i and increased 1.5x at 24 h.p.i (Figure 1F). Finally, for cells infected by the KpBSB-C isolate, CFU recovery did not present alterations (Figure 1F). These results showed that all isolates were able to invade and survive within Hep-2 cells. Furthermore, KpBSB-A and KpBSB-B also presented growth in Hep-2 cells.

All infected cells showed higher levels of NO at 24 h.p.i. indicating the activity of microbicide mechanisms by Hep-2 cells (Figure 1G). Together with invasion assays (Figure 1F) these results suggest that all isolates are resistant to these microbicidal cell mechanisms.

### Antimicrobial susceptibility test

Antimicrobial susceptibility tests were performed for 38 different antibiotic disks according to the Clinical & Laboratory Standards Institute (CLSI) (4) and the results showed extensively drug resistant (XDR) isolates (5), remaining susceptible only to aminoglycosides (amikacin, gentamicin, tobramycin, streptomycin and kanamycin). Minimum inhibitory concentration (MIC) was determined for polymyxin B and colistin by the microdilution method in a 96-well plate according to the CLSI (4), and the MIC values obtained for the three isolates were 0.5 μg/mL for both antibiotics, indicating that the isolates were susceptible to them.

### PCR screening for antimicrobial resistance genes and molecular typing methods

Initial PCR screening for antimicrobial resistance genes (6, 7) showed isolates producing *bla*_KPC_ and *bla*_SHV_ genes. Plasmid-mediated colistin resistance genes, *mcr-1* and *mcr-2*, were not found (Table 1). According to multilocus sequence typing (MLST) analysis (http://bigsdb.pasteur.fr/klebsiella/klebsiella.html), the isolates belonged to ST11 associated with the clonal group CG258 which is considered an international high-risk sequence type of KPC-producing *K. pneumoniae*. Enterobacterial Repetitive Intergenic Consensus-PCR (ERIC-PCR) analysis (8) showed the isolates presented 100% genetic similarity (Supplementary Material). The combination of the ERIC-PCR, MLST, and antimicrobial susceptibility results indicated that the three isolates were the same strain.

**Table 1:** Data on the virulence and resistance genes and plasmid replicons, obtained by draft genome analysis of *K. pneumoniae* isolates from the study.

**Isolate**	**Source**	**Genes found**		**Replicon**
		**Resistance**	**Virulence**	
Kp-BSB-A	Hemoculture	*bla*_SHV−11_, *bla*_KPC−2_, *fosA, sul1, sul2, dfrA1, oqxA, oqxB, qnrS1, tetA, tetD*	*ybtS, ybtX, ybtQ, ybtP, ybtA, Irp1, Irp2, ybtU, ybtT, ybtE, psn, fyuA, mrkA, mrkB, mrkC, mrkD, mrkE, mrkH, mrkI, mrkJ, iutA*	*IncA/C, CoIRNA1, IncN, IncR, IncFIB (pKPHS1)*

### Whole genome sequence

Two isolates (KpBSB-A and KpBSB-B) were submitted to next-generation sequencing on an Illumina NextSeq500 (150-bp paired-end) using the Nextera XT DNA sample preparation kit (Illumina, San Diego, CA, United States). The obtained sequences were assembled using SPAdes version 3.9.0 (9). The final assembly contained 92 scaffolds (>1,000 bp) and a total length of 5.6 Mb. The genomic sequences enabled the assignment of *K. pneumoniae* belonging to ST11, confirming our previous observations.

Resistance genes and plasmid replicons were determined *in silico* using the ResFinder and PlasmidFinder web services, (10, 11) and the Bacterial Isolate Genome Sequence Database (https://pubmlst.org/software/database/bigsdb/) was used to predict pathogenicity. Genome analysis showed that the isolates presented capsular type K64 and detected resistance genes for beta-lactam (*bla*_KPC−2_, *bla*_SHV−11_), sulfonamide (*sul*1, *sul*2), tetracycline (*tet*A*, tet*D), and trimethoprim (*dfr*A1). Also, a plasmid-mediated (*qnr*S1) and efflux pump complex (*oqx*A, *oqx*B) for quinolone resistance were detected. The following plasmid replicons were observed: IncR and ColRNAI, (IncN, IncA/C2, and IncFIB-like) (Table 1). In addition, the search for virulence factors detected genes related to the phenolate siderophore Yersiniabactin (*ybtA, ybtE, ybtP, ybtQ, ybtS, ybtT, ybtU, ybtX*), *irp*1, *irp*2 and *fyuA*. The *irp2* gene is observed in strains derived from urinary infections, bacteremia and other sources, and is considered an important virulence factor for extraintestinal infection establishment (12). Furthermore, mannose-specific adhesin subunit of type 3 fimbriae *mrk*ABCDF and *mrk*HIJ loci, able to mediate bacterial adhesion and biofilm formation for several surface structures (13) were also detected (Table 1). Previous studies have demonstrated that many strains belonging to ST11 do not present the mucoviscosity-associated genes *rpm*A/*mag*A (14)

### Statistical analysis

For bacterial blood and serum survival and for Hep-2 bacterial infection assays, the results were analyzed by ANOVA one way using Prism GraphPad software version 6. *P* ≤ 0.05 were considered significant.

## Background

*K. pneumoniae* is a Gram-negative bacilli associated with opportunistic and nosocomial infections such as pneumonia, meningitis and bacteremia. These strains are named classic *K. pnemoniae* and have a polyssacharide capsule that promotes their pathogenicty by phagocyte evasion. However, in the last decades, a new variant named hypermucousviscous *K. pneumoniae* (hmKP) has emerged and this scenario has become extremely complicated by be frequent association with abscess and devastating disseminated infections (15).

The hmKP strains produce a hypercapsule that promotes the ability of bacterial spread and a propensity for metastatic infection (1). Initially, these strains were also considered hypervirulent (named hvKP/hmKP) by being able to infect the non-immunocompromised population. Nowadays, several researches have reported non-hypervirulent hmKP strains (16).

Among hvKP/hmKP strains, hypermucousviscous phenotype is typically due to the increase of production of capsular polysaccharide mediated primarily by the presence of specific genes such as *rmp*A and *mag*A (17). K1 and K2 are the serotypes most associated with hvKP/hmKP, with K1 ST23 CC23 being considered a clonal group harboring specific virulence factors. These strains are also considered hypervirulent, primarily because of the association with the KPHPI patogenicity island and with virulence factors such as aerobactin, and salmochelin encoded by the pLVPK plasmid (17, 18). K2 isolates were also considered hypervirulent, however they are genetically more diverse (19).

Besides K1 and K2 serotypes, the virulence markers associated with hvKP/hmKP strains are *rmp*A, *rmp*A2, aerobactin, yersiniabactin, *pld*1, KpnO porin and a higher content of capsular sialic acid. Despite hvKP/hmKP not belonging to K1 or K2 and/or not harboring the *rmp*A/*rmp*A2 genes, they represent a puzzle regarding the genetic factors responsible for their particular phenotype (16). At the same time, evidence suggests that hmKP strains may harbor other virulence factors that promote their survival and spread in the host (16).

The prevalence of antibiotic resistance in hmKP and hvKP/hmKP strains is rare compared with the high prevalence presented by classic *K. pneumoniae* isolates (15, 17). However, reports of antibiotic-resistant hmKP and hvKP/hmKP are increasing worldwide and multidrug-resistance has been reported in Asia, Europe and America (17). Difficulties on managing carbapenem-resistant hmKP and hmKP/hvKP infections could turn these strains into the next worldwide “superbug” (17). Here we characterized three hmKP KPC-XDR isolates from a patient with bacteremia after a sepsis due to hemicolectomy surgery. The bacteremia was fatal and the isolates were obtained from blood, nasal and anal swabs. The results here obtained can contribute to understanding of the pathogenic mechanisms related to hmKP.

## Discussion

Sepsis is a common complication observed after colectomy surgery among elderly persons (20). An implication of this scenario is surgical site infections, where *K. pneumoniae* is one of the most likely organisms to be encountered (21). These infections are successfully treated by antibiotic therapy, however the presence of resistant or multidrug resistant strains can be associated with bloodstream bacterial dissemination (22). Here we reported a case of a sepsis episode in a 60–70-year-old patient, after a hemicolectomy surgery followed by bacteremia caused by a KPC-2 XDR *K. pneumoniae* strain.

Four days after presenting sepsis the patient remained non-febrile and clinically stable and the sepsis episode was considered resolved. However, the bacterial infection showed high progression and after 15 days, and *K. pneumoniae* were isolated from blood, nasal and anal cultures. All isolates were identified as KPC-XDR, ST11, and presented the same ERIC-PCR profile (Supplementary Material) and hypermucousviscous phenotype, suggesting that the bacteremia was caused by one strain presenting high spread potential.

The anastomotic dehiscence and enteric fluid in the peritoneal cavity indicate that the source of bacteremia could be associated with the surgical site infection due to the colectomy, where the XDR-KPC strain could be present in the intestine of the patient. On the other hand, the pulmonary purulent infection in the orotracheal tube developed after mechanical ventilation suggests that bacteremia could be acquired due to hospital equipment. KPC infections have been reported in hospitals in Brazil, including those located in the geographic area of the isolates (23). However, we cannot confirm these hypotheses because we do not have previous bacterial culture from the patient and/or other isolates studied from the ICU hospital.

Previous case reports from bacteremia caused by XDR *K. pneumoniae* strains showed that these infections were mostly observed in elderly patients, mainly among those exposed to invasive procedures harboring malignant and chronic diseases (24–26). As observed in our report, the strains belonged to high spreading STs such as ST258 (26) and ST11 (25), which we frequently reported as causing bloodstream infections (BSI). MLST analysis showed that the isolates belonged to ST11 CG258. ST11 is a high-risk clone associated with KPC dissemination and is commonly found in Brazil. This ST has been associated with dissemination of KPC-KP by hmKP and hvKP strains from different regions of Brazil (27). In our case report, we also identified several virulence markers, primarily iron capitation systems frequently associated with hypervirulent *K. pneumoniae* (15). The association between the risk of KPC and multidrug resistance dissemination makes this ST even more worrying.

XDR- *K. pneumoniae* BSI are considered a huge health concern because of the low or absent options for treatment. Genomic analysis showed that strains harbored beta-lactamases (SHV), carbapemases (KPC-2) and genes conferring resistance to fosfomicin (fosA), sulfamtoxazole (*sul*1, *sul*2), trimethoprim (*dfrA*1), tetracycline (*tet*Db) and to quinolones *(oqxA, oqxB,: qnrS1)* (Table 1). This analysis confirmed the resistance profile detected by antimicrobial susceptibility: that bacteremia was caused by an XDR *K. pneumoniae*, and aminoglycosides could be the unique option for therapy. However the negative result obtained from the first blood culture and the patient's clinical stability had masked the KPC-XDR infection. Therefore, meropenen prescription was continued and may contribute to the spread of infection. For sepsis, clinical symptoms frequently manifest in the absence of a positive culture resulting in diagnosis delay (28). As observed here, negative culture contributed to the wrong antibiotic prescription and was fatal to the patient. Periodical blood collection for bacterial culture could be an approach used for similar cases in order to detect silent infections, mainly in areas with previous KPC *K. pneumoniae* reports.

For BSI, monotherapy has been considered low efficacy and antibiotic combinations have shown better effectiveness due to synergic action (29). Therefore, carbapenem monotherapy should be avoided, manly if its resistance was previously reported in the hospital. Triple combination with polymyxin-colistin has shown improved efficacy to carbapenamases-producing *K. pneumoniae* infections treatment (29). On the other hand the hypercapsule of the isolates could be an obstacle to this approach since the increase of capsular polysaccharide production has been associated with polymyxin susceptibility reduction (30).

All isolates were classified as K64, a capsular serotype not yet reported with hypervirulence. Furthermore, these strains did not present several molecular virulence markers associated with hvKP strains (16). However, all isolates presented abilities to invade epithelial cells, to resist their microbicidal mechanisms, and showed cytotoxic potential (Figures 1C–E). These features suggest that the isolates harbor factors that promote epithelial host cells colonization and infection dissemination. Additionally, it has been shown that this strain may carry different virulence determinants from those already described for hmKP/hvKP. More characterization is needed to identify whether the strain here studied is also an hvKP with pathogenicity mechanisms not yet described.

The diagnosis difficulties after a sepsis episode, the high pathogenic potential presented by the isolates, the antibiotic resistance promoted by genes and by hypercapsule production, and the patient's debility contributed to the fatal character of the bacteremia. Moreover, the high prevalence of KPC-2 in Brazil demonstrates the high spread and colonization capacity presented by these strains (31). Considering all these variants, immunization programs for high-risk patients can be a precious tool to decrease the fatal potential of XDR-KPC *K. pneumoniae*. Successful immunization approaches such as the Egyptian vaccine offered broad spectrum coverage of more than 85% to all tested isolates (32) and can be a good alternative to improve the outcome for a high risk patient.

## Concluding remarks

In summary, we described a fatal case of a hypermucousviscous KPC-2 producing XDR ST11 *K. pneumoniae*, which occurred in Brazil. Resistance to carbapenems and KPC-2 are extensively reported in *K. pneumoniae* isolates from this region (30). As shown by the antimicrobial susceptibility test results, the use of aminoglycosides could have been a good therapeutic choice. However, the initial patient clinical stability, after meropenem treatment for sepsis control, may have hidden the bacteremia progress by XDR-*K. pneumoniae*. Moreover, the virulence may have also contributed to the bacteremia evolution. Periodical blood collection for bacterial culture could be an approach used for similar cases in order to detect silent infections, mainly in areas with previous KPC *K. pneumoniae* infection reports.

This Whole Genome Shotgun project has been deposited in ENA (European Nucleotide Archive) under the accession number PRJEB24576.

## Ethics statement

Ethical approval was received from the Faculdade de Ciências Farmacêuticas de Ribeirão Preto, Universidade de São Paulo, Ribeirão Preto, SP, Brazil [approval no:CEP/FCFRP 362; CAEE 36031914.9.0000.5403] and from the Faculdade de Medicina, Universidade de Brasília, Brasília, DF, Brazil [approval no. CEP/FMUnB 1.131.054;CAEE: 44867915.1.0000.558]. Extensive efforts have been made to obtain consent for the publication of this case report from the next of kin; however, as this could not be obtained, this case report has been thoroughly de-identified.

## Author contributions

TC and AP-S conceived, designed the experiments and wrote the paper. LG and VP performed blood and serum survival assays. LG and KM performed Hep-2 cell infection assays. GG, JF, and ES performed the antimicrobial susceptibility tests, PCR and molecular typing methods. GP, JP, and GPJ performed the whole genome sequence analysis.

### Conflict of interest statement

The authors declare that the research was conducted in the absence of any commercial or financial relationships that could be construed as a potential conflict of interest.

## Supplementary material

Supplementary Material Agarose gel (1.5% w/v) electrophoresis with ERIC-PCR (Enterobacterial Repeats Intergenic Consensus Sequences–PCR) profile. **1:** 1 Kb Ladder (Gibco); **2:** BSB-A ERIC-profile; **3:** BSB-B ERIC-profile; **4:** BSB-C ERIC-profile.
