# Supplementary figures and images for: A Fatal Bacteremia Caused by Hypermucousviscous KPC-2 Producing Extensively Drug-Resistant K64-ST11 Klebsiella pneumoniae in Brazil
Source: Front Med (Lausanne). 2018 Sep 21;5:265. doi: 10.3389/fmed.2018.00265 (PMC6161680; doi:10.3389/fmed.2018.00265)

LD BSB-A BSB-BBSB-C

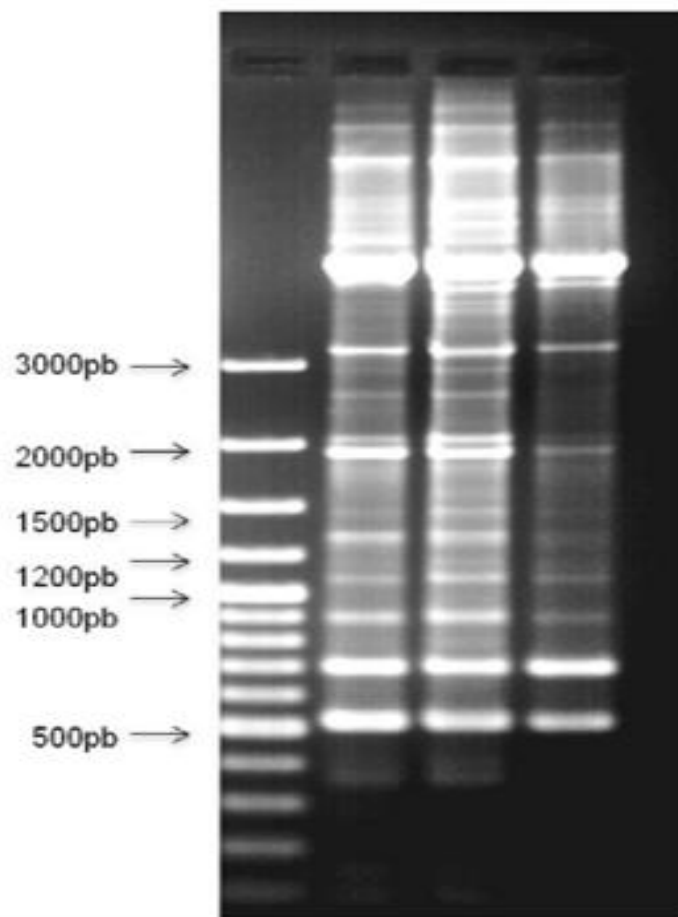

LD: bp Ladder (1Kb – Invitrogen)

Supplement: Supplementary Material — Agarose gel (1.5% w/v) electrophoresis with ERIC-PCR (Enterobacterial Repeats Intergenic Consensus Sequences–PCR) profile. 1: 1 Kb Ladder (Gibco); 2: BSB-A ERIC-profile; 3: BSB-B ERIC-profile; 4: BSB-C ERIC-profile. [file Image_1.PDF]
